# Care Processes and Clinical Responses to Newly Detected Albuminuria: The Stockholm Creatinine Measurements (SCREAM) Project

**DOI:** 10.1053/j.ajkd.2025.09.020

**Published:** 2025-12-06

**Authors:** Antoine Créon, Anne-Laure Faucon, Aurora Caldinelli, Jung-Im Shin, Morgan E. Grams, Arvid Sjölander, Edouard L. Fu, Juan-Jesus Carrero

**Affiliations:** Department of Medical Epidemiology and Biostatistics, Karolinska Institutet (ACr, A-LF, ACa, AS, ELF, J-JC), and Division of Nephrology, Department of Clinical Sciences, Danderyd Hospital (J-JC), Stockholm, Sweden; Department of Epidemiology, Bloomberg School of Public Health, Johns Hopkins University, Baltimore, Maryland (J-IS); Department of Medicine and Department of Population Health, Grossman School of Medicine, New York University, and Langone Health, New York, New York (MEG); and Department of Clinical Epidemiology, Leiden University Medical Center, Leiden, the Netherlands (ELF).

## Abstract

**Rationale & Objective:**

Albuminuria is a predictor of adverse health outcomes. Early detection enables timely clinical management, yet little is known about how clinicians respond to newly detected albuminuria in routine practice. This study characterized clinical care processes for patients with newly detected albuminuria.

**Study Design:**

Retrospective, population-based cohort study.

**Setting & Participants:**

215,035 adults with newly detected albuminuria between 2010 and 2021 in Stockholm, Sweden.

**Exposure:**

Albuminuria severity, categorized as moderate (≥30–299 mg/g), severe (300–999 mg/g), or very severe (≥1000 mg/g). All methods of albuminuria testing were considered: dipstick albuminuria or proteinuria tests as well as 24-hour and spot albumin concentrations.

**Outcome:**

Proportion of patients retested for albuminuria, frequency of the methods used for retesting, rates of nephrology referral, and rates of initiation of treatment with renin angiotensin system or sodium/glucose cotransporter 2 inhibitors.

**Analytical Approach:**

Descriptive analysis of proportions and cumulative incidence of outcomes based on time-to-event analysis accounting for the competing risks of death and kidney failure.

**Results:**

We found 90% of participants had moderate, 8% had severe, and 2% had very severe albuminuria. Retesting rates within 1 year were 46%, ranging from 45% for moderate albuminuria to 70% for very severe albuminuria, with lower rates among individuals without diabetes. Only 28% of those with an indication were referred to a nephrologist, and renin angiotensin system and sodium/glucose cotransporter 2 inhibitor initiation rates at 1 year were 10%, 12%, and 37% for moderate, severe, and very severe albuminuria, respectively, with substantially lower rates in individuals without diabetes.

**Limitations:**

The findings are specific to Stockholm’s health care system and may not be generalizable to other regions, health care models, or cultures.

**Conclusions:**

This study identified important care gaps in the Swedish management of albuminuria. A substantial proportion of individuals, including those with very severe albuminuria, lacked monitoring and failed to receive antiproteinuric treatments. Strategies to improve clinician awareness and adherence to guideline-recommended care may mitigate the long-term consequences of chronic kidney disease progression.

## PLAIN-LANGUAGE SUMMARY

Early signs of kidney damage, such as albuminuria (protein in the urine) may not always lead to the appropriate clinical follow-up that is recommended in clinical practice guidelines. This study characterized how patients with albuminuria were managed in Stockholm’s health care environment. Between 2010 and 2021, 215,035 adults had elevated albuminuria detected for the first time, but many did not receive the recommended follow-up care, including a confirmation test, referral to a nephrologist when indicated, or initiation of kidney-protective medications. This study highlights the need for better strategies to improve care for individuals with albuminuria, ensuring earlier intervention that may prevent more severe health consequences of early indications of kidney damage.

## Graphical Abstract

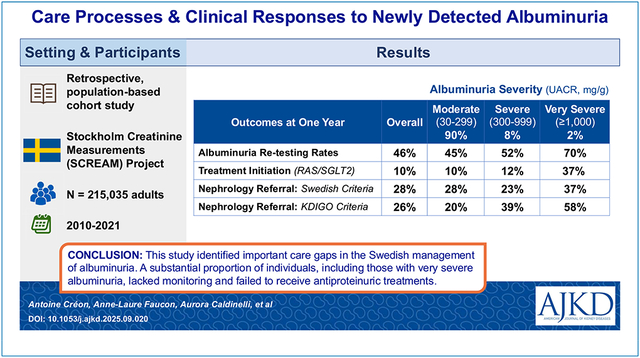

Chronic kidney disease (CKD) is expected to emerge as one of the leading causes of death worldwide by 2040, making early detection essential for effective and timely management.^1^ Albuminuria is a critical component in the diagnosis of CKD that is associated with multiple adverse outcomes, such as all-cause mortality, kidney failure with replacement therapy (KFRT), and cardiovascular disease.^2^ When elevated albuminuria is sustained over time, the guidelines recommend nephrology referral for high-risk individuals and the use of medications that provide cardio- and kidney-protective effects, such as renin angiotensin system (RAS) inhibitors and sodium/glucose cotransporter 2 (SGLT2) inhibitors.^3–5^

Investigating the quality of albuminuria care in clinical practice is important to identify system inefficiencies and address potential gaps in care. Some studies, predominantly from North American settings and with a focus on diabetes management, have documented that patients at risk of CKD are neither sufficiently screened nor monitored for albuminuria.^6–11^ Incident albuminuria may lead to higher rates of RAS inhibitor initiation, but many patients remain untreated.^10,12^ Evidence from European health systems is scarce,^13,14^ and it is unclear whether identified gaps in US practice (where access to care may be influenced by variations in insurance coverage, out-of-pocket costs, and service availability^15^) can be generalizable. Evaluation of care processes at the moment of new detection of albuminuria may circumvent limitations of previous reports and provide a clearer reflection of the ways in which systems or providers respond.

We conducted a study in Stockholm’s health care system to comprehensively assess the clinical response to the detection of incident albuminuria. We examined retesting, nephrology referral, and pharmacological management stratified by albuminuria severity and history of diabetes, and we assessed consistency across subgroups defined by age, cardiovascular comorbidities, and sex as well as over time.

## Methods

This study follows RECORD reporting guidelines.^16^

### Data Source

We used data from the Stockholm Creatinine Measurements (SCREAM) project, a health care utilization cohort including all residents of the Stockholm region in Sweden.^17^ SCREAM contains longitudinal health data from 3.2 million individuals from 2006 to 2021. Using unique personal identity numbers, SCREAM was linked to other regional and national administrative databases, which include information on demographics, socioeconomic status, health care utilization, laboratory tests, dispensed drugs, diagnoses, and vital status. The study was approved by the regional ethics review board in Stockholm and by the Swedish National Board of Welfare (#2017/793–31). Because data were linked and deidentified by the Swedish government, informed consent was not deemed necessary.

### Study Population

We identified all adults (≥18 years) with incident albuminuria, defined as an elevated urinary albumin-creatinine ratio (UACR) ≥ 30 mg/g or albuminuria detected by other methods between January 1, 2010, and December 31, 2021. All methods of albuminuria testing were considered: dipstick albuminuria or proteinuria tests, 24-hour and spot albumin concentrations, urinary protein-creatinine ratio (UPCR), and UACR. UPCR and dipstick tests were approximated as UACR values using previously validated equations.^18^ We then categorized albuminuria as moderate (≥30–299 mg/g), severe (300–999 mg/g), or very severe (≥1,000 mg/g). When different tests were performed on the same day, we prioritized them in the following order: UACR > UPCR > spot albuminuria > dipstick albuminuria. The index date was set at the first test indicating elevated albuminuria. The absence of an elevated albuminuria test before the index date was checked retrospectively back to 2006. We excluded individuals who had KFRT (ie, dialysis or kidney transplantation) before the index date.

A subcohort was formed for individuals with confirmed elevated albuminuria on a second test within 18 months. To avoid immortal time bias, the index date for the subcohort was shifted to the date of confirmed albuminuria (Fig S1).

### Covariates

Study covariates included age, sex, estimated glomerular filtration rate (eGFR), comorbidities, ongoing medications (all detailed in Table S1), and indicators of specialized care at the index date. A look-back period of 1 year was used to define baseline creatinine, and eGFR was calculated using the revised Lund-Malmö equation,^19^ which is automatically reported in Swedish health systems and was shown to be the most accurate creatinine-based equation in our population.^20^ We identified comorbidities through clinical diagnosis codes without a look-back period and by whether patients were identified in primary care or under the care of nephrologists, endocrinologists, or cardiologists (collectively termed hereafter as “specialized care”).

Medication use was defined by at least 1 record of dispensation at a Swedish pharmacy within 6 months before baseline. Educational attainment was obtained by linkage with the national labor market registry^21^ and categorized as compulsory school, secondary school, and university education. We classified the severity of CKD using KDIGO G categories based on index eGFR.^3^ Finally, we assessed whether individuals met the 2012 KDIGO^22^ or Swedish criteria^23^ for nephrology referral (Table S2).

### Study Outcomes

We evaluated key steps in albuminuria care using time-to-event analyses for the following: albuminuria and eGFR retesting, nephrology referral (in eligible individuals without prior specialist care), and initiation of antiproteinuric medication (RAS inhibitor or SGLT2 inhibitor). To better understand treatment patterns, we performed 2 complementary analyses: (1) initiation rates among those not on treatment at baseline and (2) overall use after 12 months, including both prevalent users and initiators. Patients were followed until administrative censoring (December 31, 2021), date of KFRT, death, or emigration from the Stockholm region. In the subcohort, for individuals who started treatment before albuminuria confirmation, the time-to-event was set to 1 day to reflect early initiation.

### Statistical Analysis

Descriptive statistics for categorical variables were presented as count (percentage) and for continuous variables as median (1st quartile to 3rd quartile, Q1–Q3). For time-to-event outcomes, the Aalen-Johansen estimator was used to estimate cumulative incidence functions, considering death and KFRT as competing risks. For time trend analyses, cohorts were stratified on calendar year of albuminuria detection.

As a sensitivity analysis, we repeated our main analysis after excluding albuminuria that may have been done under suspicion of an infection (ie, we excluded tests that had a urinary tract infection diagnosis or a positive leukocyte esterase within a week before or after the test) or under suspicion of hematuria in women of premenopausal age (ie, we excluded tests that had a hematuria test within a week before or after the test in women younger than 65 years). Analyses were performed using R software (R Project for Statistical Computing), version 4.4.2.^24–26^

## Results

### Characteristics at Baseline and History of Monitoring

**Figure 1.**
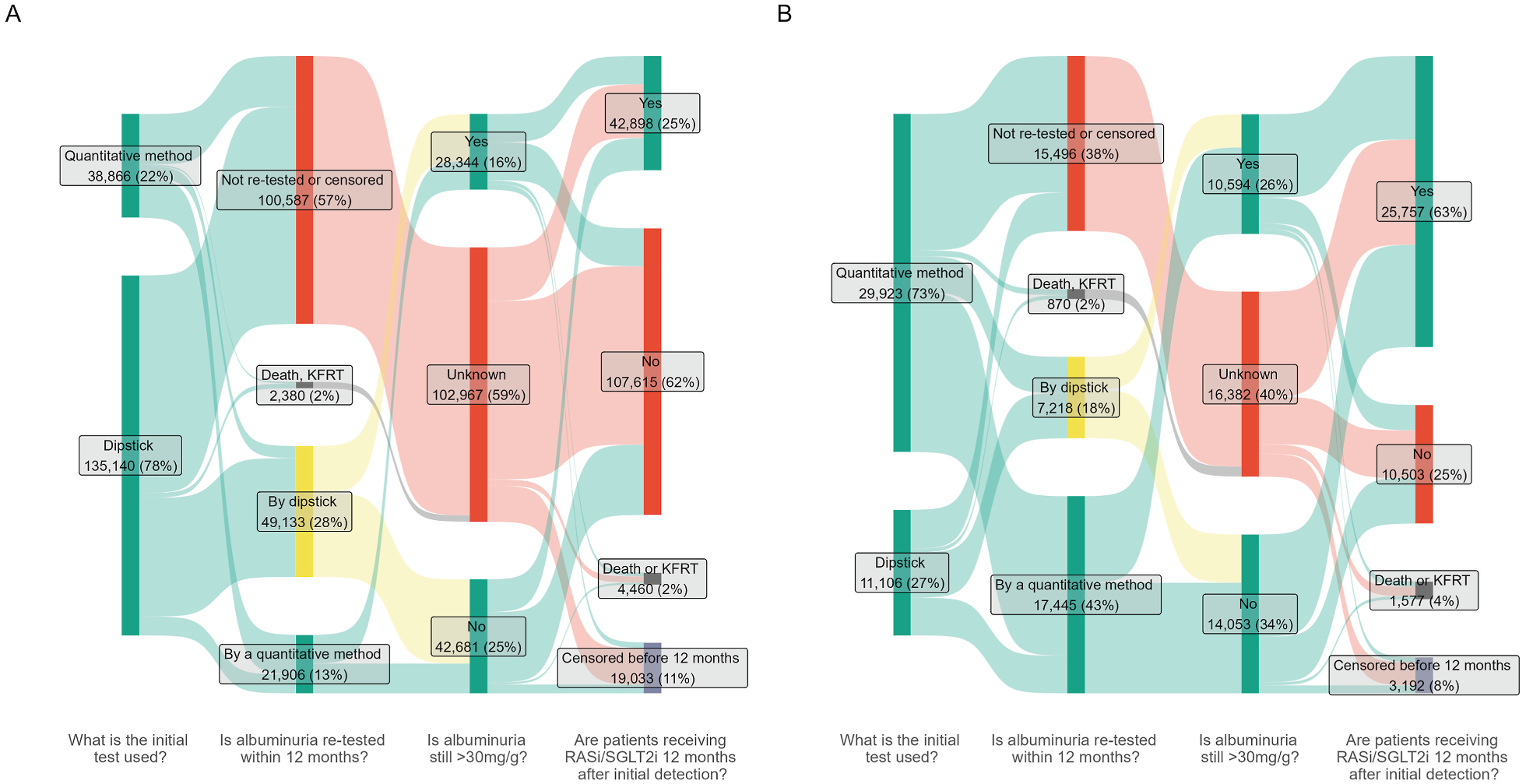
Trajectories of 1 year of care in individuals with albuminuria > 30 mg/g (A) without diabetes or (B) with diabetes. Among 215,035 individuals with newly detected elevated albuminuria, including (A) 174,006 without diabetes and (B) 41,029 with diabetes, the proportions are shown of (1) patients identified using a semiquantitative (dipstick) or quantitative method (urinary albumin-creatinine ratio, urinary protein-creatinine ratio, or 24-hour urine collection), (2) patients who underwent retesting and the method used, (3) patients with sustained elevated albuminuria (>30 mg/g) when a confirmation test was performed, and (4) patients treated with a RAS inhibitor or SGLT2 inhibitor or who experienced KFRT or death 12 months after initial albuminuria detection. Abbreviations: KFRT: kidney failure with replacement therapy; RASi, renin angiotensin system inhibitor; SGLT2i, sodium/glucose cotransporter 2 inhibitor.

Among 1,087,449 residents in the region of Stockholm that received at least 1 albuminuria test between 2010 and 2021, a total of 215,035 individuals met the inclusion criteria and had a first outpatient albuminuria measurement equivalent to UACR ≥ 30 mg/g (Fig S2), with no previous record of elevated albuminuria since 2006. Of these, 90% had moderate albuminuria (30–299 mg/g), 8% had severe albuminuria (300–999 mg/g), and 2% had very severe albuminuria (≥1,000 mg/g). Slightly more than half of the participants were women (56%), and the median age was 58 (IQR, 37–73) years. The majority (64%) had an eGFR > 60 mL/min/1.73 m^2^, and 29% were receiving a RAS inhibitor or SGLT2 inhibitor. Higher albuminuria values were associated with older age, male sex, and greater comorbidity (Table 1). Dipstick testing was the method of identification for 78% of cases in individuals without diabetes, and quantitative UACR was the method of choice for 73% of cases in individuals with diabetes (Fig 1).

At baseline, the majority (85%) of participants had a serum creatinine test within the previous 18 months, but only 35% had a documented negative albuminuria test within 18 months. Older patients were more likely to have had both albuminuria and eGFR monitored before baseline. Only 58% of individuals with diabetes had undergone an albuminuria test within the past 18 months, and preceding albuminuria monitoring rates were even lower among those with a history of hypertension (45%) or cardiovascular disease (47%) (Table S3). Most cases were identified in primary care, and a minority of participants were seen by nephrologists, cardiologists or endocrinologists. Patients seen by specialists had more comorbid conditions and rates of preceding albuminuria testing, especially among those seen by cardiologists (64%) (Tables S3–4).

### Albuminuria and eGFR Retesting

The median follow-up time was 4.8 (IQR, 2.5–7.4) years. During the year subsequent to the incident detection of elevated albuminuria, retesting with a second albuminuria test was performed in 46% of participants, ranging from 45% in those with moderate albuminuria to 70% in those with very severe albuminuria (Table S5).

**Figure 2.**
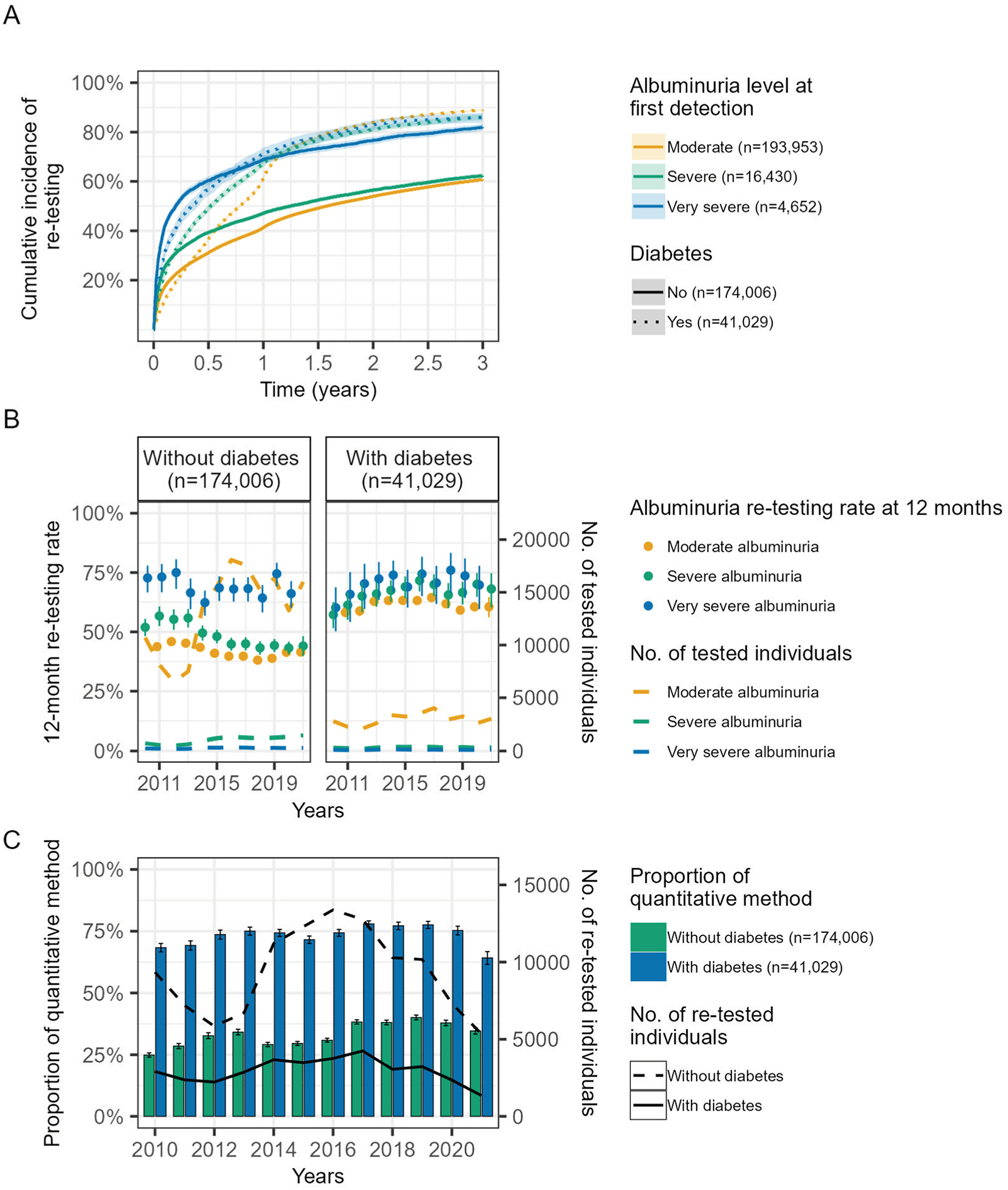
Evaluation of albuminuria retesting patterns. This analysis includes 215,035 individuals with newly detected albuminuria in Stockholm, Sweden. (A) The 3-year cumulative incidence of albuminuria retesting by categories of baseline albuminuria and history of diabetes. (B) Proportion of individuals undergoing retesting within 1 year over time (period 2010–2021). (C) Proportion of quantitative method used for retesting over time. The cumulative incidence of retesting was estimated while accounting for the competing risks of death and kidney failure with replacement therapy.

In individuals with diabetes, the 1-year retesting rates were similar across all albuminuria categories (Fig 2A; Table S5). Retesting was more frequent among older individuals, men, those with history of cardiovascular disease or hypertension, those more frequently seen in primary care, and those seen by nephrologists, endocrinologists and especially cardiologists (Table S5). Dipstick remained the most common method for albuminuria retesting in individuals without diabetes, and quantitative methods were more common in individuals with diabetes (Fig 1).

Within 3 years, 39% and 11% of patients without and with diabetes, respectively, had not received albuminuria monitoring; 3% had died (n = 7,125) or progressed to KFRT (n = 16) (Fig 2A). The retesting rates remained unchanged when excluding individuals in whom the albuminuria test may have been done under suspicion of a urinary tract infection or hematuria in women under 65 years (Table S6). Among those who received a second albuminuria test within a year, 26% with diabetes and 16% without diabetes had persistent albuminuria (Fig 1). Patients who underwent a confirmatory test and had sustained elevated albuminuria were older and had a greater burden of comorbidities (Table S7).

Retesting of eGFR within 12 months from elevated albuminuria detection was performed in 67% of individuals, with higher rates observed in older individuals, men, those with comorbid cardiovascular disease, diabetes, or hypertension, and in those with a history of specialized care or initially tested with a quantitative method (Table S5).

### Referral to Nephrology Care

Among the individuals without a history of nephrology care (n = 209,786), 5,795 (2.8%) and 5,365 (2.6%) met the Swedish and 2012 KDIGO criteria for nephrology referral at baseline, respectively (Fig S2). Overall, the 1-year rates of a first nephrology visit were low, ranging from 23% to 37% across albuminuria categories for the participants meeting the Swedish referral criteria, and from 20% to 58% for the participants meeting the KDIGO referral criteria (Table 2).

In individuals with sustained albuminuria at retesting, 3,688 (8.1%) and 6,707 (14.8%) of participants met Swedish and KDIGO criteria for nephrology referral, respectively (Fig S2). Although still low, the 1-year rates of attendance at a nephrology visit were higher, ranging from 32% to 66% across albuminuria categories for the participants meeting the Swedish referral criteria, and from 25% to 44% for the participants meeting the KDIGO referral criteria. Participants who were identified through quantitative methods were more likely to be referred to nephrology care than those identified through dipstick testing (Table 2).

### Pharmacologic Management in Patients With Newly Detected Elevated Albuminuria

**Figure 3.**
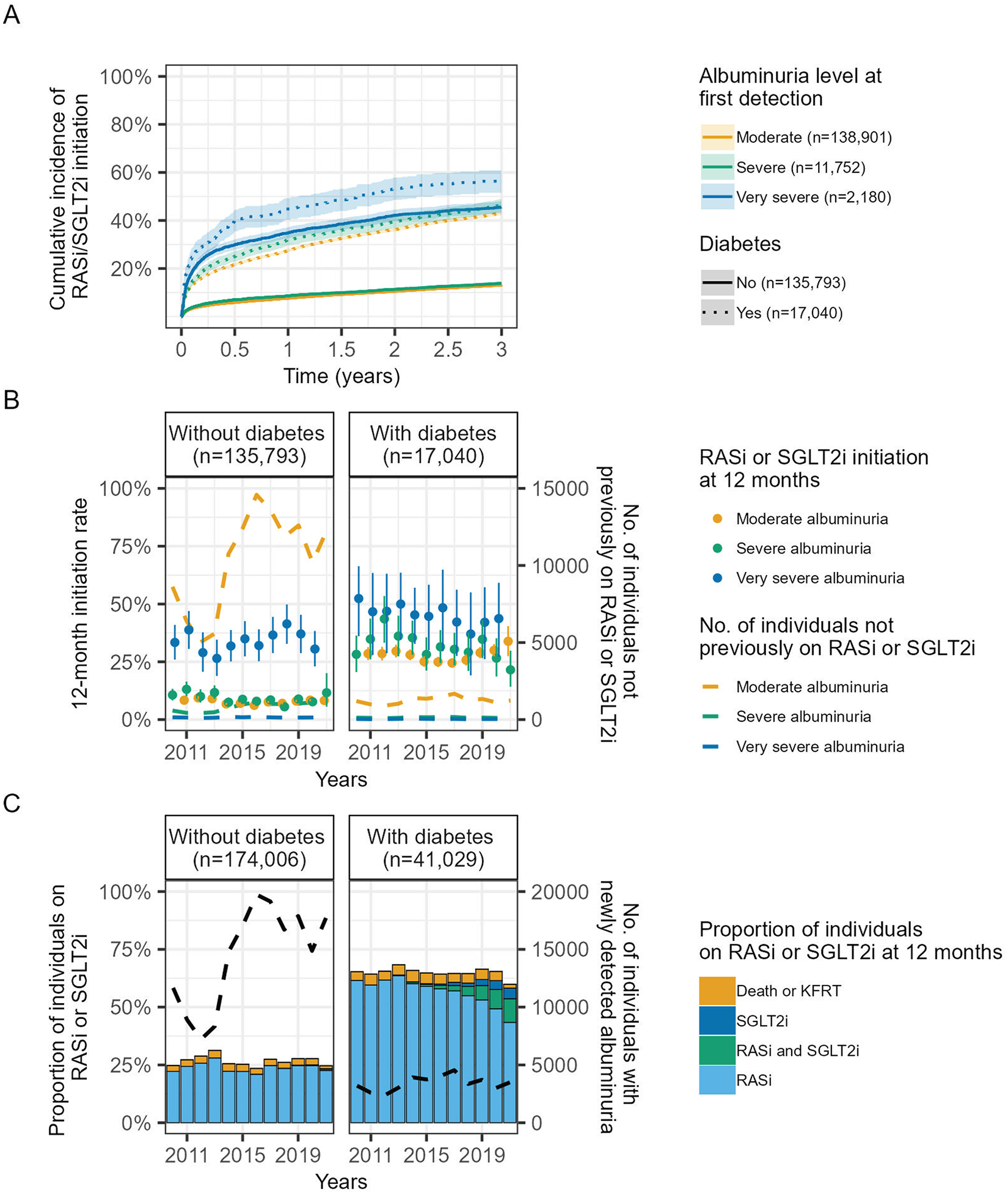
Evaluation of albuminuria management patterns. This analysis includes 215,035 individuals with newly detected albuminuria in Stockholm, Sweden. (A) The 3-year cumulative incidence of RAS inhibitor/SGLT2 inhibitor initiation by categories of baseline albuminuria and history of diabetes among previously untreated patients (n = 152,833). (B) Proportion of individuals initiating antiproteinuric treatment within 1 year over time (period 2010–2021); (C) Total population receiving RAS inhibitor/SGLT2 inhibitor within 1 year of albuminuria detection, combining prevalent and new users. The 12-month proportion of death and KFRT, consistently below 4% and 1%, respectively, are not shown. The cumulative incidence of treatment initiation was estimated while accounting for the competing risks of death and KFRT. Abbreviations: KFRT, kidney failure with replacement therapy; RASi, renin angiotensin system inhibitor; SGLT2i, sodium/glucose cotransporter 2 inhibitor.

Some patients (n = 62,202, 29%) were already receiving antiproteinuric medication at time of albuminuria detection (index date). However, the majority (n = 152,833; 71%) had never received such treatment previously. Among the participants who had not received antiproteinuric treatment, the 1-year cumulative incidence of RAS inhibitor or SGLT2 inhibitor initiation was generally low, particularly in individuals without diabetes (Fig 3A). However, the treatment rates were comparatively higher in those with diabetes (even at lower levels of albuminuria) and increased with albuminuria severity.

The initiation rates in individuals without diabetes were 8%, 9%, and 35% in the moderate, severe, and very severe albuminuria groups, respectively, compared with 27%, 32%, and 45% in those with diabetes (Fig 3A; Table S8). Less than half of participants (45%) with diabetes and very severe albuminuria were taking a RAS inhibitor or SGLT2 inhibitor at 1 year (Fig 3A; Table S8). In most cases, the first initiated agent was a RAS inhibitor, accounting for 95% (n = 30,998) of treatment initiations, compared with 5% (n = 1,443) for SGLT2 inhibitors.

### Time Trends in Care Processes After Newly Detected Albuminuria

Each calendar year, between 9,586 and 23,772 individuals had newly detected elevated albuminuria, with an increase observed during the early study period (2010–2015). Although retesting rates among individuals with diabetes also rose during this time, other trends—including the cumulative incidence of retesting at 12 months in those without diabetes, the method used for retesting, and initiation of a RAS inhibitor or SGLT2 inhibitor—remained stable over time (Fig 2B and C, and Fig 3B). Antiproteinuric treatment use at 12 months was substantially higher in individuals with diabetes compared with those without (around 60% vs 25%). Although SGLT2 inhibitor dispensation after elevated albuminuria detection progressively rose in individuals with diabetes, this did not translate to a higher overall proportion of treated patients (Fig 3C).

### Pharmacologic Management in Patients With Confirmed Elevated Albuminuria

**Figure 4.**
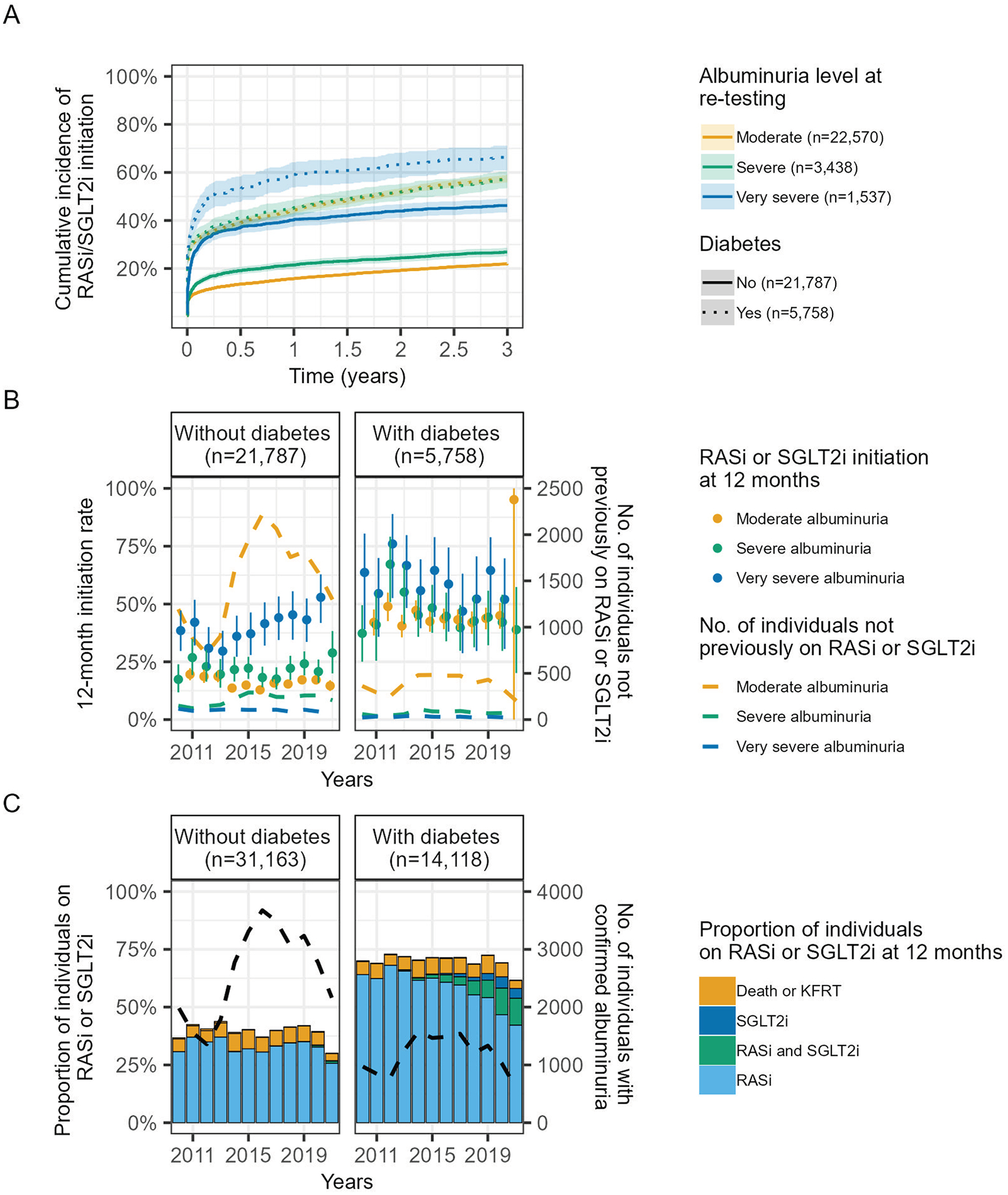
Evaluation of albuminuria management patterns in individuals with confirmed albuminuria. This analysis includes 45,281 individuals with confirmed albuminuria (ie, 2 consecutive elevated albuminuria tests) in Stockholm, Sweden. (A) Cumulative incidence of RAS inhibitor/SGLT2 inhibitor initiation among previously untreated individuals (n = 27,545). (B) Proportion of individuals initiating antiproteinuric treatment within 1 year over time (period 2010–2021). (C) Total population receiving RAS inhibitor/SGLT2 inhibitor within 1 year of albuminuria confirmation, combining prevalent and new users. The 12-month proportion of death and KFRT, consistently below 4% and 1%, respectively, are not shown. The cumulative incidence of treatment initiation was estimated while accounting for the competing risks of death and KFRT. Abbreviations: KFRT, kidney failure with replacement therapy; RASi, renin angiotensin system inhibitor; SGLT2i, sodium/glucose cotransporter 2 inhibitor.

In the subcohort of individuals with sustained albuminuria at retesting, the initiation of a RAS inhibitor or SGLT2 inhibitor was more rapid than in those with a single abnormal test, ranging from 1 year in 22% participants with moderate albuminuria to 44% in those with very severe albuminuria, with similar trends in those with newly detected albuminuria (Fig 4A; Table S9). Still, in 34% and 54% of participants with confirmed severe albuminuria, with or without diabetes, respectively, no antiproteinuric treatment was initiated within 3 years of retesting (Fig 4A). The initiation rates were lower in women, in individuals younger than 65 years, and in those with CKD stages 1–2 with moderate and severe albuminuria. Time trend analyses were consistent with those observed after a single test (Fig 4B and C).

## Discussion

In this large study of individuals with newly detected elevated albuminuria in Stockholm’s health care system, we observed important gaps in care processes that persisted over time: albuminuria retesting rates were low and were primarily performed using an insensitive semiquantitative method. In addition, a large number of individuals did not have their albuminuria levels confirmed or followed over time, did not receive antiproteinuric medication, or were not referred to a nephrologist for care despite meeting the guideline-recommended criteria. These differences persisted across high-risk groups and among individuals with confirmed albuminuria at retesting.

Clinical guidelines recommend retesting for albuminuria at least 3 months after detection to confirm that the elevated levels are sustained over time.^22^ In our study, this was performed in 46% of cases within 1 year. Although low, the albuminuria retesting rates observed in our study were higher than those previously reported. In Canada, only 39% of patients with CKD stage G3–5 managed in primary care underwent retesting within 6 months after an abnormal UACR test.^10^ In the United States, 6.7% of individuals with an abnormal dipstick result were retested within a year.^9^ A potential explanation of our higher retesting rates is that, although previous studies focused on either UACR or dipstick testing, our study provided a more comprehensive assessment of follow-up practices by considering all methods of albuminuria testing jointly. Of note, dipstick testing was the most frequently used retesting method for individuals without diabetes in our study, despite its lower reliability compared with quantitative methods.^27^ In general, the retesting rates were higher for individuals with diabetes, which may reflect the long-standing recommendations by KDIGO and the American Diabetes Association guidelines to monitor albuminuria annually.^22,28^ This speculation is supported by the convergence of retesting rates across albuminuria levels at 1 year as well as by previously reported high retesting rates even among individuals with diabetes who had a negative initial test.^9^

Only 1 in 4 patients who met the criteria for nephrology referral in our study were seen by a nephrologist within 1 year. This proportion is considerably lower than the 55% referral rate reported in a US study of insured patients with CKD.^11^ Several factors may contribute to this discrepancy: whereas the US study primarily included individuals with reduced eGFR, our cohort consisted predominantly of patients with preserved kidney function, for whom referral may be perceived as less urgent. Additionally, differences in health care systems and referral pathways may play a role, emphasizing the need to better understand barriers to nephrology care even in a universal health care setting.

A disappointingly low number of patients (24%) with confirmed albuminuria were receiving antiproteinuric medications 1 year after detection, a finding echoed in other studies. Bello et al^10^ reported that only 30.5% of patients with confirmed CKD and proteinuria in Canada were prescribed a RAS inhibitor within 1 year. In a US study, 35.6% and 43.1% of individuals with newly UACR-detected moderate and severe albuminuria, respectively, initiated treatment within 6 months, and overall initiation was as low as 5% in those detected by dipstick.^12^

Similar to the US study,^12^ we show that undertreatment may disproportionately affect women, younger patients, and those with preserved kidney function. The potential explanations may include perception of risk and differences in physician prescribing patterns.^29^ Our findings further demonstrate that, although overall initiation rates were low, they were consistently higher—though still suboptimal—in individuals with diabetes. Again, we speculate that higher initiation rates may be a consequence of adopting guideline recommendations such as KDIGO 2012, which supported treatment of moderate albuminuria only in individuals with diabetes. Given that dipstick testing was the predominant method of albuminuria detection in our study, this may also have contributed to therapeutic inertia: clinicians might be less likely to act on semiquantitative results or may question the reliability of an initial positive test.

Evaluating time trends in clinical practice is valuable for several reasons, including the tracking of quality improvement or the effectiveness of new policies and/or interventions. We are not aware of prior studies addressing this issue. Although we observed a progressive increase in the use of SGLT2 inhibitors after 2017, this did not result in an overall increase in the proportion of patients being monitored or receiving treatment for albuminuria. It is plausible that those who eventually received a SGLT2 inhibitor were the same individuals who had previously been appropriately managed with a RAS inhibitor and albuminuria monitoring, suggesting that SGLT2 inhibitor uptake occurred within a subgroup whose care was already guideline adherent. It is also possible that SGLT2 inhibitors were prescribed as a replacement for RAS inhibitors rather than as an addition, or that their initiation was driven by glycemia management rather than albuminuria. Although albuminuria screening increased during the early years of the study, as reflected by the rising number of detected cases, other aspects of albuminuria care remained largely unchanged over time. Collectively, this persistence suggests that therapeutic improvements, increased disease awareness, educational initiatives, and related policies have not translated into quantifiable improvements in primary care.

Qualitative research has explored reasons behind this inertia. Many general practitioners (GPs) view early-stage CKD as complex^30,31^ or may not recognize the impact of moderate albuminuria in patients with eGFR ≥ 60 mL/min/1.73 m^2^ as CKD.^32^ Uncertainty may be compounded by the variety of urine tests and thresholds, each with different costs and indications.^33–35^ Some GPs may find annual albuminuria monitoring impractical, are unfamiliar with newer kidney-protective therapies, or may prioritize more immediate health concerns.^35^ Others perceive nephrology referral as offering limited added value.^30,35^ Patient-level factors also contribute: CKD awareness is generally low, which may reduce follow-up engagement.^36,37^ Primary care settings face structural challenges because they serve populations with high rates of multimorbidity, polypharmacy, and aging. In such contexts, quality of life and patient preferences may appropriately take precedence over strict adherence to disease-specific guidelines.

In the Stockholm region, a unified health care system with shared electronic health records ensures laboratory data are accessible across providers. Despite this infrastructure—which could facilitate systematic follow-up of abnormal results—our findings reveal substantial gaps in albuminuria-related care. Possibly infrastructure alone is insufficient without active follow-up systems and clinician engagement. Educational initiatives targeting both physicians and patients could reinforce the value of albuminuria testing in assessing kidney and cardiovascular risk. Clear and concise guidelines for GPs should highlight the benefits of early detection and intervention. Policy interventions, such as pay-for-performance programs, have improved albuminuria testing rates in countries like France^38^ and the United Kingdom,^34,39^ though these must be carefully designed to avoid narrowing testing to incentivized groups.^34,35,40^ Strengthening collaboration between nephrologists and GPs through comanagement models has improved CKD care in other settings,^41,42^ as have clinical decision support tools that integrate kidney-specific recommendations.^42–47^ Although structured programs to improve CKD care have been limited in Stockholm, recent efforts—including new clinical decision support systems and educational initiatives for physicians—may change the scenario presented here in the coming years. Future research should evaluate the drivers of the observed disparities, notably between sexes, and the impact of these interventions on care delivery and patient outcomes, including the consequences of delayed treatment initiation.

The main strengths of our study include its comprehensive evaluation of processes of care in individuals with newly detected albuminuria. We may offer more granularity than previous studies by capturing all health care provided in our region and all methods of albuminuria assessment. We also see as a study strength the evaluation of a complete northern European health system with universal tax-funded health care, which may minimize ascertainment biases due to health care fragmentation or disparities in access to care or affordability of medications.

Our study also has limitations. First, we cannot assess clinician reasoning, such as why certain patients were not referred or treated, nor account for potential contraindications (eg, hypotension) or patient refusal to undertake specific tests, take prescribed medications, or attend nephrologist care. Second, we could not account for potential point-of-care albuminuria testing; although it is available in Sweden, it is rarely performed because only laboratory-based tests are reimbursed. This may have led to misclassification. Finally, our study represents the clinical practice in the region of Stockholm. Extrapolation to other regions, countries, health care models, or care cultures should be done with caution. However, given that our findings are in line with previous North American reports, we believe that the reported suboptimal care in our study is not a problem exclusive to Sweden.

To conclude, this study highlights critical gaps in the care processes of patients with albuminuria. Despite the availability of effective medications, a substantial proportion of individuals, including those with very severe albuminuria, lacked monitoring and failed to receive antiproteinuric treatments. Strategies are urgently needed to ensure that albuminuria is systematically recognized and treated as a key component of CKD management.

## Supplementary Material

1 **Figure S1:** Graphical depiction of the study design.
**Figure S2**: Study flowchart.
**Table S1**: Definition of study covariates and outcomes.
**Table S2**: Nephrology referral criteria.
**Table S3:** History of albuminuria and eGFR monitoring before index date.
**Table S4:** Baseline characteristics of individuals with newly detected albuminuria, by history of specialized follow-up.
**Table S5:** Cumulative incidence of creatinine and albuminuria retesting within 12 months after newly detected albuminuria.
**Table S6:** Cumulative incidence of albuminuria retesting within 12 months after newly detected albuminuria, when excluding results suspicious of urinary tract infection or hematuria in women of premenopausal age.
**Table S7:** Baseline characteristics of people with confirmed elevated albuminuria, overall and by albuminuria level.
**Table S8:** Cumulative incidence at 12 months of RAS inhibitor or SGLT2 inhibitor use after first elevated albuminuria detection in previously untreated individuals.
**Table S9:** Cumulative incidence at 12 months of RAS inhibitor or SGLT2 inhibitor use after confirmed elevated albuminuria in previously untreated individuals.

Supplementary File (PDF)

## Figures and Tables

**Table 1.** Baseline Characteristics of Individuals With Newly Detected Albuminuria, Overall and by Albuminuria Level

		Albuminuria Level		
Demographics	Overall N = 215,035	Moderate N = 193,953 (90%)^a^	Severe N = 16,430 (8%)^b^	Very Severe N = 4,652 (2%)^c^
Age, y	58 [37–73]	57 [37–72]	57 [36–73]	68 [54–78]
Age group				
<65	129,040 (60%)	117,176 (60%)	9,980 (61%)	1,884 (40%)
65–75	39,882 (19%)	35,795 (18%)	2,870 (17%)	1,217 (26%)
>75	46,113 (21%)	40,982 (21%)	3,580 (22%)	1,551 (33%)
Sex				
Male	94,152 (44%)	84,432 (44%)	6,342 (39%)	3,378 (73%)
Female	120,883 (56%)	109,521 (56%)	10,088 (61%)	1,274 (27%)
Highest educational attainment				
Compulsory school	43,724 (21%)	39,109 (21%)	3,454 (22%)	1,161 (26%)
Secondary school	86,951 (42%)	78,600 (42%)	6,454 (41%)	1,897 (42%)
University	76,980 (37%)	69,798 (37%)	5,770 (37%)	1,412 (32%)
**Comorbidities**				
Hypertension	88,480 (41%)	78,581 (41%)	6,470 (39%)	3,429 (74%)
Cardiovascular disease	33,779 (16%)	29,703 (15%)	2,725 (17%)	1,351 (29%)
Heart failure	14,743 (7%)	12,755 (7%)	1,325 (8%)	663 (14%)
Diabetes mellitus	41,029 (19%)	35,530 (18%)	4,124 (25%)	1,375 (30%)
Recent cancer, 3 y	16,512 (8%)	14,410 (7%)	1,415 (9%)	687 (15%)
Liver disease	6,381 (3%)	5,647 (3%)	527 (3%)	207 (4%)
CKD diagnosis	1,279 (1%)	1,057 (1%)	142 (1%)	80 (2%)
**Clinical Health Care**				
Seen by nephrologist	5,249 (2%)	4,437 (2%)	489 (3%)	323 (7%)
Seen by endocrinologist	18,536 (9%)	16,574 (9%)	1,455 (9%)	507 (11%)
Seen by cardiologist	73,235 (34%)	65,745 (34%)	5,560 (34%)	1,930 (41%)
No. of primary care visits in the previous year				
0	28,016 (13%)	25,273 (13%)	2,303 (14%)	440 (9%)
1	30,575 (14%)	27,740 (14%)	2,335 (14%)	500 (11%)
2–4	63,284 (29%)	57,437 (30%)	4,598 (28%)	1,249 (27%)
5–9	48,559 (23%)	43,784 (23%)	3,670 (22%)	1,105 (24%)
≥10	44,601 (21%)	39,719 (20%)	3,524 (21%)	1,358 (29%)
**Clinical and Laboratory Measurements**				
eGFR, mL/min/1.73 m^2^	77 [62–91]	77 [62–91]	74 [58–90]	63 [46–79]
eGFR KDIGO category				
G1–2	137,713 (64%)	125,846 (65%)	9,379 (57%)	2,488 (53%)
G3a	25,018 (12%)	22,232 (11%)	1,899 (12%)	887 (19%)
G3b	10,520 (5%)	9,013 (5%)	971 (6%)	536 (12%)
G4	4,416 (2%)	3,454 (2%)	547 (3%)	415 (9%)
G5	521 (0%)	282 (0%)	127 (1%)	112 (2%)
Unknown	36,847 (17%)	33,126 (17%)	3,507 (21%)	214 (5%)
Albuminuria, mg/g	53 [38–184]	53 [38–111]	664 [380–721]	1,221 [1,009–1,621]
Type of albuminuria test				
24-h urine albumin excretion	484 (0%)	327 (0%)	81 (0%)	76 (2%)
Dipstick	146,246 (68%)	131,484 (68%)	12,113 (74%)	2,649 (57%)
UACR	64,473 (30%)	58,580 (30%)	4,039 (25%)	1,854 (40%)
Urine albumin concentration	3,832 (2%)	3,562 (2%)	197 (1%)	73 (2%)
**Medications**				
β-Blocker	48,366 (22%)	42,409 (22%)	4,019 (24%)	1,938 (42%)
Calcium channel blocker	36,893 (17%)	32,467 (17%)	2,877 (18%)	1,549 (33%)
Thiazide diuretic	5,038 (2%)	4,483 (2%)	376 (2%)	179 (4%)
ACE inhibitor/ARB	61,857 (29%)	54,737 (28%)	4,651 (28%)	2,469 (53%)
SGLT2 inhibitor	1,067 (0%)	956 (0%)	83 (1%)	28 (1%)
MRA	4,575 (2%)	3,994 (2%)	386 (2%)	195 (4%)

Values are given as median [IQR] for continuous variables and as number (percentage) for categorical variables. Abbreviations: ACE, angiotensin-converting enzyme; ARB, angiotensin 2 receptor antagonist; CKD, chronic kidney disease; MRA, mineralocorticoid receptor antagonist; SGLT2, sodium/glucose cotransporter 2; UACR, urinary albumin-creatinine ratio.

a Moderate albuminuria: 30–299 mg/g.

b Severe albuminuria: 300–999 mg/g.

c Very severe albuminuria: >1,000 mg/g.

**Table 2.** Cumulative Incidence of Nephrology Referral Within 12 Months From Albuminuria Detection, Among Individuals Meeting Referral Criteria

	After Newly Detected Elevated Albuminuria					After Confirmed Elevated Albuminuria				
			Albuminuria Level					Albuminuria Level		
	No. at Risk	Overall^a^	Moderate^b^	Severe^c^	Very Severe^d^	No. at Risk	Overall^a^	Moderate^b^	Severe^c^	Very Severe^d^
Swedish criteria^e^	5,795	28% (27–30)	28% (26–29)	23% (21–25)	37% (34–39)	3,688	42% (40–43)	32% (30–34)	44% (41–48)	66% (62–69)
Dipstick^f^	2,831	11% (10–12)	15% (13–17)	7% (6–9)	9% (7–12)	1,470	22% (20–24)	15% (13–18)	24% (19–29)	43% (37–50)
Quantitative method^f^	2,964	45% (43–47)	37% (35–40)	44% (41–48)	64% (60–68)	2,218	55% (53–57)	45% (42–48)	56% (51–60)	76% (72–79)
KDIGO criteria^g^	5,365	26% (24–27)	20% (19–21)	39% (35–42)	58% (53–62)	6,707	32% (31–33)	30% (29–32)	25% (23–27)	44% (42–47)

a 12-Month cumulative incidence (95% CI).

b Moderate albuminuria: 30–299 mg/g.

c Severe albuminuria: 300–999 mg/g.

d Very severe albuminuria: ≥1,000 mg/g.

e Based on a combination of age, albumin-creatinine ratio, and eGFR thresholds (see Table S2).

f Type of test at first detection for individuals with newly detected albuminuria, and type of test at albuminuria confirmation for individuals with confirmed albuminuria.

g Estimated GFR < 30 mL/min per 1.73 m^2^ or refractory hypertension after newly detected albuminuria; eGFR < 30 mL/min per 1.73 m^2^, refractory hypertension or sustained albuminuria ≥ 300 mg/g at retesting.

## Data Availability

The data underlying this article cannot be shared publicly due to concerns about participant privacy. However, data may be made available upon reasonable request to Prof. Carrero (juan.jesus.carrero@ki.se) for academic research collaborations that comply with the General Data Protection Regulation (GDPR) as well as national and institutional ethics regulations and standards.
