# Supplementary material for: Care Processes and Clinical Responses to Newly Detected Albuminuria: The Stockholm Creatinine Measurements (SCREAM) Project
Source: Am J Kidney Dis. Author manuscript; Available in PMC 2026 Jun 23. (PMC13287621; doi:10.1053/j.ajkd.2025.09.020)
Supplement: 1 — Figure S1: Graphical depiction of the study design. Figure S2: Study flowchart. Table S1: Definition of study covariates and outcomes. Table S2: Nephrology referral criteria. Table S3: History of albuminuria and eGFR monitoring before index date. Table S4: Baseline characteristics of individuals with newly detected albuminuria, by history of specialized follow-up. Table S5: Cumulative incidence of creatinine and albuminuria retesting within 12 months after newly detected albuminuria. Table S6: Cumulative incidence of albuminuria retesting within 12 months after newly detected albuminuria, when excluding results suspicious of urinary tract infection or hematuria in women of premenopausal age. Table S7: Baseline characteristics of people with confirmed elevated albuminuria, overall and by albuminuria level. Table S8: Cumulative incidence at 12 months of RAS inhibitor or SGLT2 inhibitor use after first elevated albuminuria detection in previously untreated individuals. Table S9: Cumulative incidence at 12 months of RAS inhibitor or SGLT2 inhibitor use after confirmed elevated albuminuria in previously untreated individuals. [file NIHMS2187451-supplement-1.pdf]

Figure S1: Graphical depiction of the study Design

This graphical depiction follows the framework suggested by Schneeweiss et al., *Annals of Internal Medicine*, 2019[1] and is adapted from templates available at www.repeatinitiative.org/projects.html.

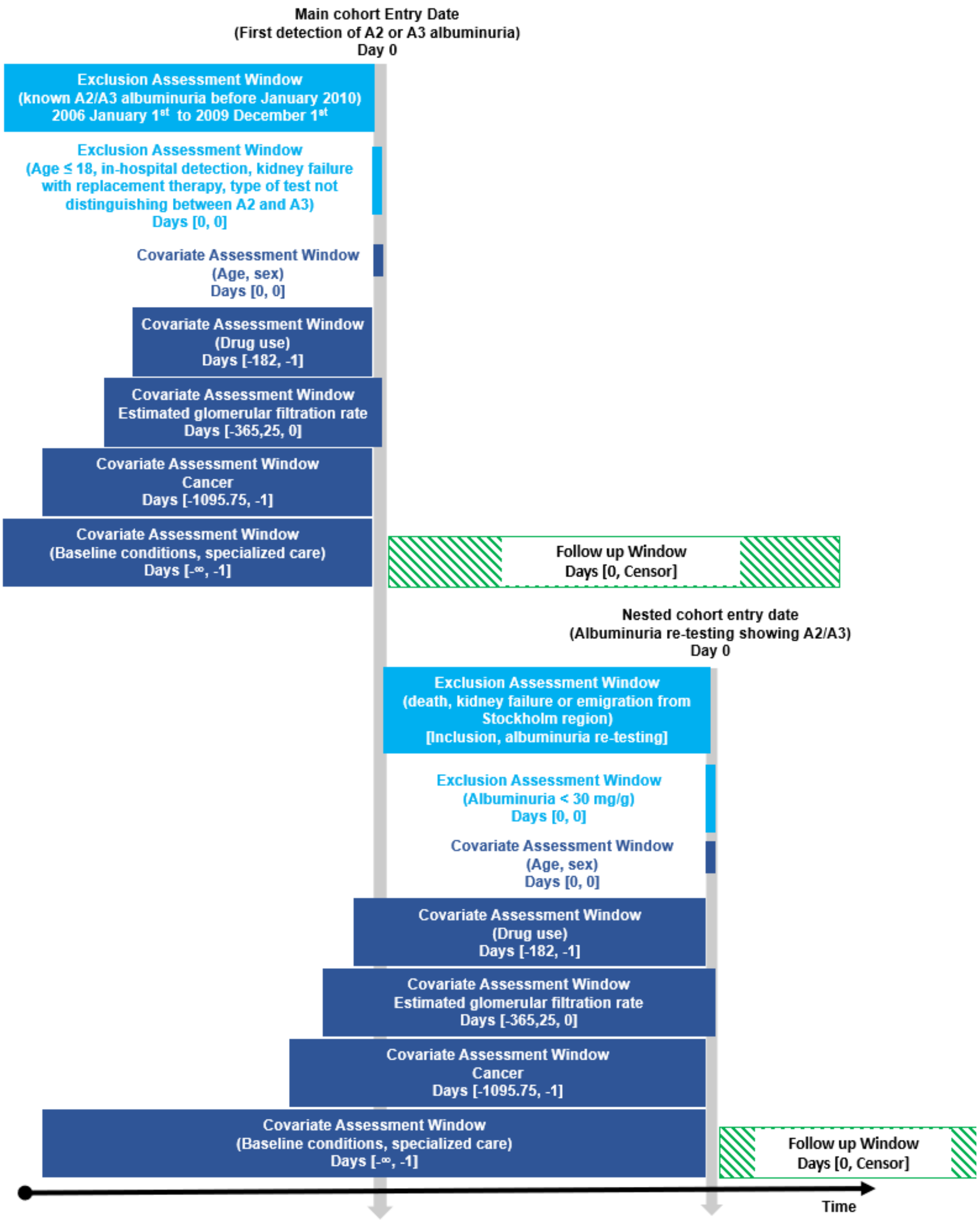

Figure S2: Study Flowchart

*Swedish referral criteria are defined in Table S2

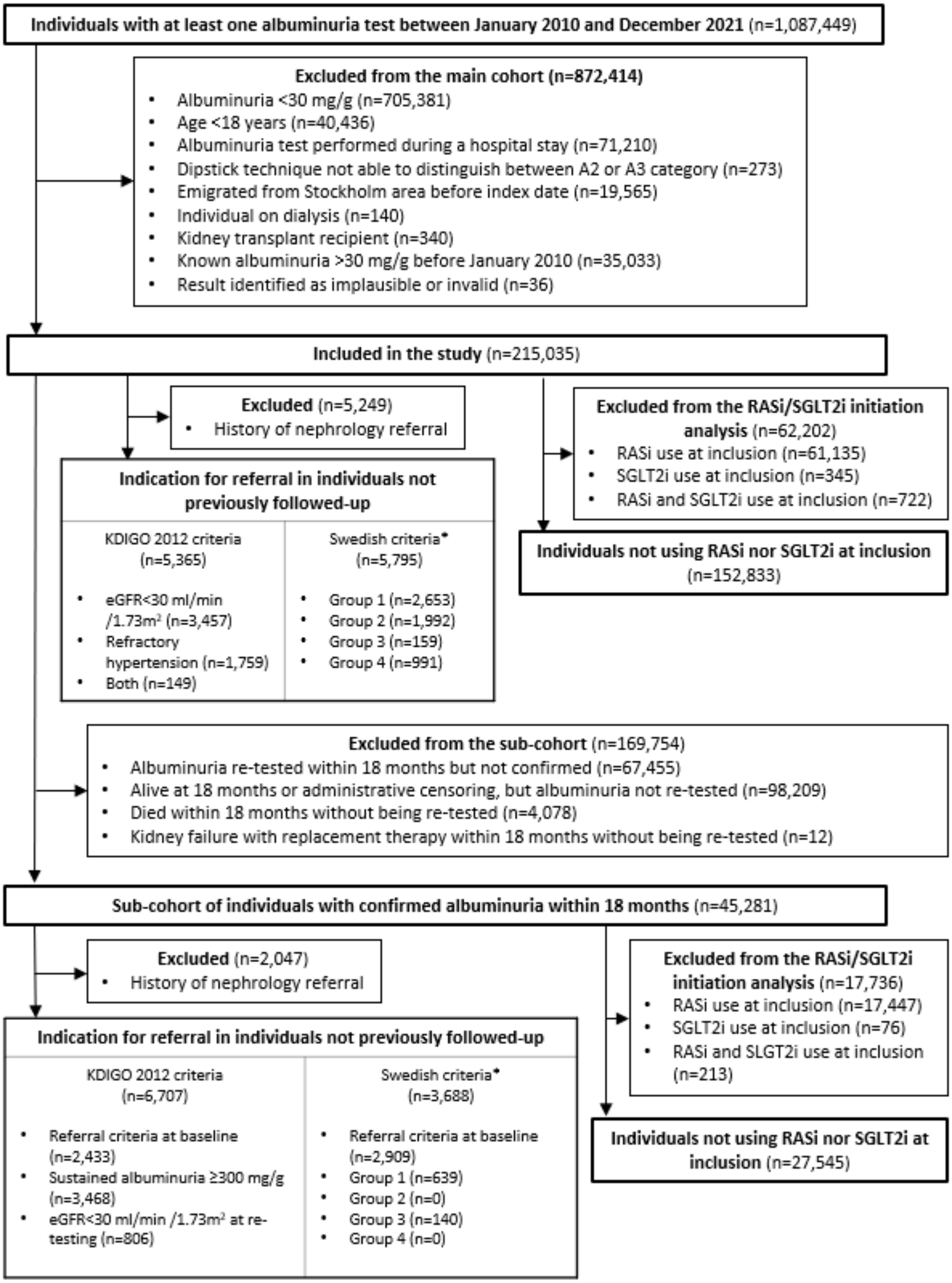

Table S1: Definition of study covariates and outcomes.

Individuals were considered active drug users if a drug dispensation was recorded within 6 months before index date.

Variables	Codes (ICD-10 or ATC)
**Comorbidities**	
Cardiovascular disease	I200, I21, I22, I201, I208, I209, I24, I25, I60, I61, I62, I63, I64, I693, I698, I694, I70, I72, I73
Hypertension	I10-I15
Heart failure	I110, I130, I132, I42, I43, I50
Diabetes	E10-E14
Cancer	C00-C26, C30-C34, C37-C41, C43-C58, C60-C76, C81-C96, C97
Liver disease	B18, I850, I859, I982, K70-K77
Chronic kidney disease	E112, I131, I120, I132, N032, N033, N034, N035, N036, N037, N052, N053, N054, N055, N056, N057, N18, N19, N250, Z490, Z491, Z492, Z940, Z992
**Medications**	
Beta-blockers	C07
Calcium channel blockers	C08
Thiazide diuretics	C03A
Renin-angiotensin system inhibitors	C09A, C09B, C09C, C09D, C09DX04
Sodium-glucose cotransporter-2 inhibitors	A10BK, A10BD15-16, A10BD19-21, A10BD23-25, A10BD27, A10BD29
Mineralocorticoid receptor antagonists (steroidal or not)	C03DA
Peripherally-acting alpha-blockers	C02CA
Centrally-acting alpha-blockers	C02AC
Pyrimidine derivative (Minoxidil)	C02DC
Neprilysin inhibitor	C09DX04

Table S2: Nephrology referral criteria

**Swedish criteria**			
Group	Age (years)	Albumin-creatinine ratio (mg/g)	eGFR (ml/min per 1.73 m2)
1	<75	<300	<45
2	<75	≥300	<60
3	≥75	<700	<15
4	≥75	≥700	<50
**KDIGO 2012 criteria**			
eGFR <30 ml/min/1.73 m^2			
Confirmed albuminuria ≥300 mg/g			
Refractory hypertension*			

*Hypertension refractory to treatment was defined by the presence of filled prescriptions for 4 or more different antihypertensive agents in the 6 months before the index date.

Table S3: History of albuminuria and eGFR monitoring before index date

	Overall		Albuminuria level					
			Moderate*		Severe†		Very severe‡	
	Albuminuria testing	eGFR testing	Albuminuria testing	eGFR testing	Albuminuria testing	eGFR testing	Albuminuria testing	eGFR testing
Overall	35% (34-35)	85% (85-86)	35% (35-35)	85% (85-86)	31% (30-32)	82% (81-82)	38% (36-39)	97% (96-97)
Age group								
<65	29% (28-29)	78% (78-78)	29% (29-29)	78% (78-78)	25% (24-26)	73% (72-74)	33% (31-36)	96% (95-97)
65-75	43% (43-44)	96% (96-96)	44% (43-44)	96% (96-96)	42% (40-44)	94% (93-95)	41% (38-44)	97% (96-98)
>75	44% (44-44)	98% (98-98)	44% (44-45)	98% (98-98)	41% (39-42)	97% (96-97)	40% (37-42)	97% (96-98)
Sex								
Male	35% (34-35)	92% (92-92)	35% (34-35)	92% (92-92)	34% (33-35)	93% (92-94)	38% (36-40)	97% (96-97)
Female	35% (34-35)	80% (80-80)	35% (35-36)	80% (80-81)	30% (29-30)	75% (74-76)	36% (34-39)	96% (95-98)
Diabetes								
No	29% (29-30)	82% (82-82)	30% (29-30)	82% (82-82)	24% (24-25)	76% (76-77)	33% (32-35)	96% (95-97)
Yes	58% (57-58)	99% (99-99)	59% (58-60)	99% (99-99)	52% (50-53)	98% (98-98)	47% (45-50)	98% (97-99)
Other comorbidities								
History of CKD diagnosis	67% (64-70)	100% (99-100)	71% (68-73)	100% (99-100)	51% (42-59)	99% (96-100)	48% (36-59)	99% (92-100)
Cardiovascular disease	47% (46-47)	98% (98-98)	47% (47-48)	98% (98-98)	44% (43-46)	98% (97-98)	41% (38-44)	98% (97-99)
Hypertension	46% (45-46)	98% (98-98)	46% (45-46)	98% (98-98)	44% (42-45)	97% (97-98)	41% (40-43)	98% (97-98)
Follow-up history								
Nephrology	42% (41-42)	92% (92-92)	42% (41-42)	92% (92-92)	40% (38-41)	91% (90-92)	43% (41-45)	98% (97-98)
Endocrinology	48% (48-49)	95% (95-96)	49% (48-50)	95% (95-96)	45% (43-48)	94% (93-95)	45% (40-49)	99% (97-100)
Cardiology	64% (63-66)	99% (98-99)	65% (64-67)	99% (98-99)	56% (52-60)	98% (96-99)	66% (60-71)	99% (98-100)
Primary care only	30% (29-30)	81% (80-81)	30% (30-30)	81% (80-81)	26% (25-26)	76% (75-77)	31% (29-33)	95% (94-96)
Number of primary care visits in the previous year								
0	21% (21-22)	73% (72-73)	21% (21-22)	73% (73-74)	18% (16-19)	66% (64-68)	30% (26-35)	94% (91-96)
1	21% (21-22)	75% (75-76)	21% (21-22)	75% (75-76)	17% (16-19)	70% (68-72)	28% (24-32)	93% (90-95)
2-4	31% (31-31)	83% (83-83)	31% (31-32)	83% (83-83)	27% (26-28)	78% (77-79)	34% (32-37)	96% (95-97)
5-9	42% (42-42)	91% (91-92)	42% (42-43)	91% (91-92)	39% (38-41)	90% (88-90)	40% (38-43)	97% (96-98)
≥10	50% (49-50)	97% (97-97)	50% (50-51)	97% (97-97)	47% (45-49)	97% (96-98)	44% (41-46)	99% (98-99)

*Moderate albuminuria: 30–299 mg/g
†Severe albuminuria: 300–999 mg/g
‡Very severe albuminuria: ≥1000 mg/g

Table S4: Baseline characteristics of individuals with newly detected albuminuria, by history of specialized follow-up

**Baseline Characteristics**	**Exclusive primary care N = 130,262***	**Nephrology N = 5,249***	**Endocrinology N = 18,536***	**Cardiology N = 73,235***
Age	53 (33, 70)	68 (54, 78)	63 (49, 74)	65 (45, 76)
Age group				
<65	86,244 (66%)	2,232 (43%)	9,711 (52%)	36,329 (50%)
65-75	21,516 (17%)	1,281 (24%)	4,490 (24%)	15,793 (22%)
>75	22,502 (17%)	1,736 (33%)	4,335 (23%)	21,113 (29%)
Sex				
Male	53,810 (41%)	2,956 (56%)	8,080 (44%)	35,377 (48%)
Female	76,452 (59%)	2,293 (44%)	10,456 (56%)	37,858 (52%)
Highest educational attainment				
Compulsory school	24,937 (20%)	1,209 (24%)	4,137 (23%)	16,304 (23%)
Secondary school	51,401 (41%)	2,167 (43%)	8,050 (44%)	30,650 (43%)
University	48,216 (39%)	1,721 (34%)	6,009 (33%)	24,886 (35%)
Hypertension	43,138 (33%)	3,614 (69%)	10,596 (57%)	39,355 (54%)
Cardiovascular disease	11,023 (8%)	1,498 (29%)	4,432 (24%)	21,274 (29%)
Heart failure	3,492 (3%)	1,006 (19%)	2,059 (11%)	10,706 (15%)
Diabetes mellitus	19,937 (15%)	1,325 (25%)	7,283 (39%)	17,397 (24%)
Recent cancer (3 years)	7,148 (5%)	737 (14%)	2,075 (11%)	8,352 (11%)
Liver disease	2,646 (2%)	443 (8%)	1,292 (7%)	3,096 (4%)
Chronic kidney disease diagnosis	384 (0%)	370 (7%)	307 (2%)	638 (1%)
History of nephrology referral			1,099 (6%)	2,741 (4%)
History of endocrinology referral		1,099 (21%)		9,070 (12%)
History of cardiology referral		2,741 (52%)	9,070 (49%)	
Number of primary care visits in the previous year				
0	20,017 (15%)	605 (12%)	1,467 (8%)	6,819 (9%)
1	21,660 (17%)	494 (9%)	1,614 (9%)	7,687 (10%)
2-4	41,188 (32%)	1,183 (23%)	4,430 (24%)	19,003 (26%)
5-9	27,588 (21%)	1,179 (22%)	4,787 (26%)	18,015 (25%)
≥10	19,809 (15%)	1,788 (34%)	6,238 (34%)	21,711 (30%)
eGFR, ml/min/1.73m2	79 (65, 93)	48 (29, 70)	75 (60, 89)	72 (57, 87)
eGFR KDIGO category				
G1-2	83,271 (64%)	1,867 (36%)	12,986 (70%)	46,767 (64%)
G3a	12,178 (9%)	882 (17%)	2,660 (14%)	11,391 (16%)
G3b	4,501 (3%)	1,057 (20%)	1,121 (6%)	5,186 (7%)
G4	1,548 (1%)	1,125 (21%)	528 (3%)	2,268 (3%)
G5	170 (0%)	206 (4%)	46 (0%)	222 (0%)
Unknown	28,594 (22%)	112 (2%)	1,195 (6%)	7,401 (10%)
Albuminuria, mg/g	53 (38, 184)	59 (41, 197)	57 (41, 183)	57 (41, 184)
Albuminuria category				
Moderate	117,967 (91%)	4,437 (85%)	16,574 (89%)	65,745 (90%)
Severe	9,940 (8%)	489 (9%)	1,455 (8%)	5,560 (8%)
Very severe	2,355 (2%)	323 (6%)	507 (3%)	1,930 (3%)
Type of albuminuria test				
24-hour urine albumin excretion	257 (0%)	22 (0%)	126 (1%)	135 (0%)
Dipstick	93,724 (72%)	1,939 (37%)	10,253 (55%)	46,250 (63%)
Urine albumin-creatinine ratio	34,155 (26%)	3,176 (61%)	7,701 (42%)	25,415 (35%)
Urine albumin concentration	2,126 (2%)	112 (2%)	456 (2%)	1,435 (2%)
Beta blocker	19,390 (15%)	2,292 (44%)	5,964 (32%)	26,253 (36%)
Calcium channel blocker	18,488 (14%)	1,808 (34%)	4,322 (23%)	15,746 (22%)

**Baseline Characteristics**	**Exclusive primary care N = 130,262***	**Nephrology N = 5,249***	**Endocrinology N = 18,536***	**Cardiology N = 73,235***
Thiazide diuretic	2,779 (2%)	180 (3%)	596 (3%)	1,869 (3%)
ACEi/ARB†	29,641 (23%)	2,585 (49%)	7,534 (41%)	28,027 (38%)
SGLT2 inhibitor‡	411 (0%)	21 (0%)	246 (1%)	558 (1%)
Mineralocorticoid receptor antagonist	1,190 (1%)	320 (6%)	815 (4%)	3,128 (4%)

*Median (Q1, Q3); n (%)
†Angiotensine Converting Enzyme inhibitor / Angiotensin 2 receptor antagonist
‡Sodium-Glucose cotransporter 2 inhibitor

Table S5: Cumulative incidence of creatinine and albuminuria re-testing within 12 months after newly detected albuminuria

	**Overall**		**Albuminuria level**					
			**Moderate***		**Severe†**		**Very severe‡**	
	Albuminuria re-testing§	eGFR re-testing§	Albuminuria re-testing§	eGFR re-testing§	Albuminuria re-testing§	eGFR re-testing§	Albuminuria re-testing§	eGFR re-testing§
Overall	46% (46-46)	67% (67-68)	45% (45-45)	66% (66-67)	52% (51-53)	71% (70-72)	69% (68-71)	91% (90-91)
Age group								
<65	41% (41-41)	57% (57-57)	40% (40-40)	56% (56-56)	46% (45-47)	60% (59-61)	75% (73-77)	90% (88-91)
65-75	55% (54-55)	80% (80-80)	54% (53-54)	79% (79-80)	63% (62-65)	86% (84-87)	69% (66-72)	91% (90-93)
>75	52% (52-53)	85% (85-85)	51% (51-52)	84% (84-85)	60% (59-62)	90% (89-91)	64% (61-66)	91% (89-92)
Sex								
Female	45% (44-45)	63% (63-63)	44% (44-44)	63% (62-63)	48% (47-49)	64% (63-65)	74% (72-76)	92% (91-94)
Male	48% (48-48)	73% (73-73)	46% (46-47)	71% (71-72)	59% (58-61)	82% (81-83)	68% (66-69)	90% (89-91)
Diabetes								
No	42% (42-42)	63% (63-64)	41% (41-42)	63% (62-63)	47% (46-48)	64% (63-65)	69% (67-70)	90% (89-91)
Yes	62% (62-63)	84% (83-84)	61% (61-62)	83% (82-83)	67% (66-68)	90% (89-91)	71% (69-74)	91% (89-92)
Education								
Compulsory school	49% (48-49)	74% (74-74)	48% (47-48)	73% (73-74)	56% (55-58)	78% (76-79)	70% (67-72)	90% (88-92)
Secondary school	46% (46-47)	68% (68-69)	45% (45-46)	67% (67-68)	53% (52-54)	72% (71-74)	68% (66-70)	91% (89-92)
University	44% (44-45)	63% (62-63)	43% (43-44)	62% (62-62)	49% (48-50)	65% (64-67)	71% (69-74)	91% (90-93)
Other comorbidities								
Cardiovascular disease	55% (54-55)	86% (86-86)	53% (53-54)	85% (85-86)	62% (60-63)	90% (89-91)	64% (62-67)	93% (91-94)
Hypertension	54% (54-55)	82% (81-82)	53% (53-53)	81% (81-81)	64% (63-66)	89% (88-90)	67% (65-68)	90% (89-91)
Type of test at baseline								
Dipstick	42% (41-42)	62% (61-62)	41% (41-41)	61% (61-61)	47% (46-47)	64% (63-65)	62% (60-64)	88% (87-89)
Quantitative method	56% (55-56)	79% (79-80)	54% (53-54)	78% (78-79)	68% (66-69)	89% (88-90)	79% (78-81)	94% (93-95)
Follow-up history								
Nephrology	50% (50-51)	76% (76-77)	49% (49-50)	76% (75-76)	57% (56-59)	81% (79-82)	68% (65-70)	91% (90-92)
Endocrinology	56% (55-57)	82% (81-82)	55% (54-56)	81% (80-81)	62% (60-65)	86% (84-87)	72% (68-76)	93% (91-95)
Cardiology	68% (67-69)	89% (88-90)	66% (64-67)	88% (87-89)	76% (72-79)	93% (91-95)	87% (83-90)	97% (94-98)
Primary care only	43% (42-43)	61% (61-61)	42% (41-42)	60% (60-61)	48% (47-49)	64% (63-65)	70% (68-71)	90% (88-91)
History of albuminuria monitoring								
Yes	60% (60-61)	79% (79-79)	59% (59-60)	78% (78-78)	67% (66-68)	84% (83-85)	78% (76-80)	94% (92-95)
Number of primary care visits in the previous year								
0	35% (34-36)	52% (51-52)	34% (34-35)	51% (50-52)	39% (37-41)	53% (51-55)	67% (62-71)	88% (84-91)
1	38% (37-38)	54% (53-54)	37% (36-37)	53% (52-53)	43% (41-45)	59% (57-61)	72% (67-76)	89% (86-91)
2-4	43% (43-44)	62% (62-63)	42% (42-42)	61% (61-62)	49% (47-50)	67% (65-68)	70% (67-72)	88% (86-90)
5-9	51% (50-51)	74% (73-74)	50% (49-50)	73% (72-73)	59% (57-61)	77% (76-79)	69% (66-72)	91% (89-92)
≥10	57% (56-57)	86% (85-86)	56% (55-56)	85% (85-85)	63% (62-65)	88% (87-90)	70% (67-72)	94% (93-95)

*Moderate albuminuria: 30–299 mg/g
†Severe albuminuria: 300–999 mg/g
‡Very severe albuminuria: ≥1000 mg/g
§12-month cumulative incidence (95% confidence interval), in percentage.

Table S6: Cumulative incidence of albuminuria re-testing within 12 months after newly detected albuminuria, when excluding results suspicious of urinary tract infection or hematuria in women of premenopausal age.

	Overall	Stratified by albuminuria level		
		30-299 mg/g*	300-999 mg/g*	>1000 mg/g*
Excluding women under 65 years, with hematuria >1+ (n = 3,302)				
Number of individuals	211,733	191,083	16,023	4,627
Cumulative incidence[1]	46% (46-46)	45% (45-45)	53% (52-53)	70% (68-7)
Excluding individuals with leukocyte esterase > 1+ (n=8,328)				
Number of individuals	206,707	186,669	15,554	4,484
Cumulative incidence[1]	46% (46-46)	45% (45-45)	52% (51-53)	70% (68-71)
Excluding individuals with (n=11,223)				
Number of individuals	203,812	185,053	14,620	4,139
Cumulative incidence[1]	46% (46-46)	45% (45-45)	53% (52-54)	70% (69-72)

[*]12-month cumulative incidence (95% confidence interval), in percentage.

Table S7: Baseline characteristics of people with confirmed elevated albuminuria, overall and by albuminuria level

Baseline Characteristics	Overall	Albuminuria level		
		**Moderate**	**Severe**	**Very severe**
	N=45,281*	N=36,657 (81%)†	N=5,688 (13%)‡	N=2,936 (6%)§
Age	66 (47, 77)	66 (48, 77)	66 (46, 77)	67 (46, 77)
Age group				
<65	21,209 (47%)	17,161 (47%)	2,701 (47%)	1,347 (46%)
65-75	10,262 (23%)	8,371 (23%)	1,204 (21%)	687 (23%)
>75	13,810 (30%)	11,125 (30%)	1,783 (31%)	902 (31%)
Sex				
Male	24,174 (53%)	19,331 (53%)	2,922 (51%)	1,921 (65%)
Female	21,107 (47%)	17,326 (47%)	2,766 (49%)	1,015 (35%)
Highest educational attainment				
Compulsory school	10,764 (25%)	8,648 (24%)	1,422 (26%)	694 (25%)
Secondary school	18,575 (43%)	15,108 (43%)	2,292 (42%)	1,175 (42%)
University	14,283 (33%)	11,641 (33%)	1,701 (31%)	941 (33%)
Hypertension	25,894 (57%)	20,647 (56%)	3,243 (57%)	2,004 (68%)
Cardiovascular disease	10,491 (23%)	8,227 (22%)	1,441 (25%)	823 (28%)
Heart failure	5,283 (12%)	4,063 (11%)	755 (13%)	465 (16%)
Diabetes mellitus	14,118 (31%)	11,009 (30%)	2,181 (38%)	928 (32%)
Recent cancer (3 years)	5,759 (13%)	4,544 (12%)	770 (14%)	445 (15%)
Liver disease	1,765 (4%)	1,386 (4%)	242 (4%)	137 (5%)
Albuminuria measurement in the 18 months before first detection	17,369 (38%)	14,307 (39%)	2,027 (36%)	1,035 (35%)
Chronic kidney disease diagnosis	904 (2%)	634 (2%)	163 (3%)	107 (4%)
History of nephrology referral	2,047 (5%)	1,453 (4%)	322 (6%)	272 (9%)
History of endocrinology referral	4,667 (10%)	3,690 (10%)	646 (11%)	331 (11%)
History of cardiology	17,383 (38%)	14,002 (38%)	2,203 (39%)	1,178 (40%)
Albuminuria at retesting, mg/g	83 (46, 258)	61 (41, 177)	558 (368, 721)	1,326 (1,029, 2,256)
Type of albuminuria test at baseline				
24-hour urine albumin excretion	191 (0%)	76 (0%)	37 (1%)	78 (3%)
Dipstick	22,458 (50%)	18,661 (51%)	2,694 (47%)	1,103 (38%)
Urine albumin-creatinine ratio	21,710 (48%)	17,162 (47%)	2,848 (50%)	1,700 (58%)
Urine albumin concentration	922 (2%)	758 (2%)	109 (2%)	55 (2%)
Type of albuminuria test at re-testing				
24-hour urine albumin excretion	254 (1%)	116 (0%)	43 (1%)	95 (3%)
Dipstick	22,870 (51%)	18,694 (51%)	2,991 (53%)	1,185 (40%)
Urine albumin-creatinine ratio	21,248 (47%)	17,103 (47%)	2,531 (44%)	1,614 (55%)
Urine albumin concentration	909 (2%)	744 (2%)	123 (2%)	42 (1%)
eGFR, ml/min/1.73m2	70 (53, 87)	71 (55, 88)	67 (48, 86)	60 (38, 80)
eGFR KDIGO category at re-testing				
G1-2	28,028 (62%)	23,321 (64%)	3,271 (58%)	1,436 (49%)
G3a	7,333 (16%)	5,902 (16%)	919 (16%)	512 (17%)
G3b	4,029 (9%)	3,030 (8%)	590 (10%)	409 (14%)
G4	2,644 (6%)	1,771 (5%)	481 (8%)	392 (13%)
G5	623 (1%)	319 (1%)	154 (3%)	150 (5%)
Unknown	2,624 (6%)	2,314 (6%)	273 (5%)	37 (1%)
Beta blocker	14,952 (33%)	11,611 (32%)	2,104 (37%)	1,237 (42%)
Calcium channel blocker	12,150 (27%)	9,461 (26%)	1,618 (28%)	1,071 (36%)
Thiazide diuretic	1,392 (3%)	1,128 (3%)	161 (3%)	103 (4%)
ACEi/ARB¶	19,265 (43%)	15,288 (42%)	2,449 (43%)	1,528 (52%)
SGLT2 inhibitor#	442 (1%)	359 (1%)	64 (1%)	19 (1%)
Mineralocorticoid receptor antagonist	1,452 (3%)	1,125 (3%)	207 (4%)	120 (4%)

*Median (Q1, Q3); n (%); †Moderate albuminuria: 30-299 mg/g; ‡Severe albuminuria: 300-999 mg/g; §Very severe albuminuria: >1000 mg/g; ¶Angiotensine Converting Enzyme inhibitor / Angiotensin 2 receptor antagonist; #Sodium-Glucose cotransporter 2 inhibitor

Table S8: Cumulative incidence at 12 months of RASi or SGLT2i use after first elevated albuminuria detection, in previously untreated individuals

		**Albuminuria level**		
	**Overall***	**Moderate†,***	**Severe‡,***	**Very severe§,***
Overall	10% (10-10)	10% (10-10)	12% (11-12)	37% (35-39)
Age group				
<65	7% (7-8)	7% (7-7)	9% (9-10)	44% (41-47)
65-75	19% (19-20)	19% (18-19)	19% (17-21)	36% (31-41)
>75	17% (16-17)	16% (16-17)	17% (16-19)	25% (22-29)
Sex				
Female	8% (7-8)	7% (7-7)	8% (7-8)	34% (31-37)
Male	15% (14-15)	14% (13-14)	20% (19-21)	39% (36-41)
Diabetes				
No	8% (8-8)	8% (7-8)	9% (8-9)	35% (33-37)
Yes	28% (28-29)	27% (27-28)	32% (30-34)	45% (40-49)
Other Comorbidities				
Cardiovascular disease	20% (19-21)	20% (19-20)	21% (18-23)	29% (25-33)
Hypertension	28% (28-29)	28% (27-28)	30% (28-32)	34% (31-36)
eGFR category				
G1-2	11% (11-12)	11% (11-11)	13% (12-14)	35% (32-38)
G3a	18% (18-19)	17% (17-18)	23% (20-26)	35% (30-40)
G3b	21% (20-22)	19% (18-20)	27% (23-32)	41% (34-47)
G4	22% (20-24)	18% (16-20)	23% (18-28)	54% (47-62)
G5	21% (16-26)	11% (6-17)	22% (14-32)	38% (26-49)
Unknown	2% (2-2)	2% (2-2)	3% (2-3)	33% (24-41)
Follow-up history				
Nephrology	12% (11-12)	11% (11-11)	14% (12-15)	32% (29-35)
Endocrinology	15% (14-15)	14% (13-15)	18% (15-20)	40% (33-47)
Cardiology	19% (17-20)	17% (15-18)	27% (22-32)	41% (33-50)
Primary care only	9% (9-10)	9% (9-9)	10% (10-11)	39% (37-42)
Referral Criteria				
KDIGO¶	22% (20-24)	18% (16-20)	23% (19-28)	50% (43-56)
Swedish#	28% (27-30)	22% (20-24)	27% (24-30)	45% (41-49)
Type of test at baseline				
Dipstick	5% (5-5)	5% (5-5)	6% (5-6)	27% (24-30)
Quantitative method	30% (30-31)	29% (29-30)	36% (34-38)	47% (44-50)

*12-month cumulative incidence (95% confidence interval), in percentage.
†Moderate albuminuria: 30–299 mg/g
‡Severe albuminuria: 300–999 mg/g
§Very severe albuminuria: ≥1000 mg/g
¶eGFR<30 ml/min per 1.73 m2 or refractory hypertension at baseline
#Based on a combination of age, albumin-to-creatinine ratio and eGFR thresholds, see Supplementary Table 2

Table S9: Cumulative incidence at 12 months of RASi or SGLT2i use after confirmed elevated albuminuria, in previously untreated individuals

		**Albuminuria level**		
	Overall*	Moderate†,*	Severe‡,*	Very severe§,*
Overall	24% (23-24)	22% (21-22)	27% (26-29)	44% (42-47)
Age group				
<65	20% (20-21)	17% (17-18)	26% (24-28)	50% (46-53)
65-75	32% (31-34)	31% (29-32)	37% (33-41)	49% (43-55)
>75	25% (24-26)	25% (24-26)	24% (21-27)	29% (25-34)
Sex				
Female	17% (17-18)	16% (15-16)	21% (19-23)	37% (33-40)
Male	31% (30-32)	28% (27-29)	36% (33-38)	51% (47-54)
Diabetes				
No	18% (17-18)	16% (15-16)	21% (20-23)	40% (37-43)
Yes	45% (44-47)	44% (43-46)	45% (42-49)	59% (54-64)
Other comorbidities				
Cardiovascular disease	31% (30-33)	31% (30-33)	29% (25-33)	38% (32-43)
Hypertension	43% (42-44)	42% (41-43)	45% (42-48)	47% (44-51)
eGFR category				
G1-2	23% (23-24)	22% (21-23)	27% (25-29)	41% (38-44)
G3a	31% (29-33)	29% (27-31)	37% (32-41)	44% (37-50)
G3b	33% (31-36)	29% (27-32)	36% (30-43)	57% (50-64)
G4	31% (29-34)	26% (23-29)	31% (25-37)	57% (49-64)
G5	26% (21-31)	20% (14-27)	30% (20-40)	33% (22-44)
Unknown	4% (3-5)	4% (3-5)	2% (1-5)	22% (9-40)
Follow-up history				
Nephrology	23% (23-24)	22% (21-23)	26% (23-28)	39% (35-44)
Endocrinology	28% (26-30)	25% (23-27)	35% (30-41)	55% (46-63)
Cardiology	31% (28-34)	27% (23-30)	38% (31-46)	47% (37-56)
Primary care only	23% (22-24)	21% (20-22)	27% (25-29)	46% (42-49)
Referral Criteria				
KDIGO¶	36% (35-38)	26% (23-28)	35% (33-37)	49% (46-52)
Swedish#	38% (36-40)	28% (26-31)	44% (40-49)	60% (55-64)
Type of test at baseline				
Dipstick	11% (11-12)	10% (9-10)	14% (13-16)	39% (35-43)
Quantitative method	43% (42-44)	42% (41-43)	44% (41-46)	47% (44-51)

*12-month cumulative incidence (95% confidence interval), in percentage.
†Moderate albuminuria: 30–299 mg/g; ‡Severe albuminuria: 300–999 mg/g; §Very severe albuminuria: ≥1000 mg/g
¶eGFR<30 ml/min per 1.73 m2, refractory hypertension at re-testing or sustained albuminuria ≥300 mg/g
#Based on a combination of age, albumin-to-creatinine ratio and eGFR thresholds, see Supplementary Table 2

References

1. Schneeweiss S, Rassen JA, Brown JS, et al. Graphical Depiction of Longitudinal Study Designs in Health Care Databases. *Ann Intern Med*. 2019;170(6):398-406. doi:10.7326/M18-3079
